# Application of a tuning-free burned area detection algorithm to the Chornobyl wildfires in 2022

**DOI:** 10.1038/s41598-023-32300-5

**Published:** 2023-03-31

**Authors:** Jun Hu, Yasunori Igarashi, Shunji Kotsuki, Ziping Yang, Mykola Talerko, Volodymyr Landin, Olha Tyshchenko, Mark Zheleznyak, Valentyn Protsak, Serhii Kirieiev

**Affiliations:** 1 Center for Environmental Remote Sensing, Chiba University, 1-33 Yayoi-cho, Inage, Chiba, 263-8522 Japan; 2 Radiation Measurement Research Group, National Institutes for Quantum Science and Technology, 4-9-1 Anagawa, Inage-ku, Chiba, 263-8555 Japan; 3 Institute of Environmental Radioactivity, Fukushima University, Fukushima, 960-1296 Japan; 4 Institute for Advanced Academic Research, Chiba University, 1-33 Yayoi-cho, Inage, Chiba, 263-8522 Japan; 5 Graduate School of Science and Engineering, Chiba University, 1-33 Yayoi-cho, Inage, Chiba, 263-8522 Japan; 6 Institute for Safety Problems of Nuclear Power Plants, National Academy of Sciences of Ukraine, 12 Lysogirska str., Kyiv, 03028 Ukraine; 7 Ukrainian Hydrometeorological Institute, National Academy of Sciences of Ukraine, 37 Nauky Ave, Kyiv, 03028 Ukraine; 8 Chornobyl Ecocentre, State Agency of Ukraine On Exclusion Zone Management, Vulytsya Shkilʹna, Chornobyl, 07270 Kyiv Oblast Ukraine

**Keywords:** Environmental impact, Natural hazards

## Abstract

The wildfires in the Chornobyl Exclusion Zone (ChEZ) have caused widespread public concern about the potential risk of radiation exposure from radionuclides resuspended and redistributed due to the fires in 2020. The wildfires were also confirmed in ChEZ in the spring of 2022, and its impact needed to be estimated accurately and rapidly. In this study, we developed a tuning-free burned area detection algorithm (TuFda) to perform rapid detection of burned areas for the purpose of immediate post-fire assessment. We applied TuFda to detect burned areas in the ChEZ during the spring of 2022. The size of the burned areas in February and March was estimated as 0.4 km^2^ and 70 km^2^, respectively. We also applied the algorithm to other areas outside the boundaries of the ChEZ and detected land surface changes totaling 553 km^2^ in northern Ukraine between February and March 2022. These changes may have occurred as a result of the Russian invasion. This study is the first to identify areas in northern Ukraine impacted by both wildfires and the Russian invasion of Ukraine in 2022. Our algorithm facilitates the rapid provision of accurate information on significant land surface changes whether caused by wildfires, military action, or any other factor.

## Introduction

Changes in land surface conditions detected by satellite imagery can provide diverse information, such as on the occurrence of wildfires or artificial disturbances. For instance, thermal anomalies detected by satellites indicated that in 2020 wildfires in the Chornobyl Exclusion Zone (ChEZ) spread over an extensive area, and satellite images helped to produce an initial estimate that the burned area covered nearly 30% of the ChEZ^1,2^. Such information was used to assess the redistribution of radionuclides^3,4^ and the potential risk of radiation exposure due to the fire and smoke^5^. Satellite images are the only information available for estimating the burned area in case of large wildfires. Accurate and rapid detection of wildfires is essential for producing reliable atmospheric model simulations of smoke and radionuclide resuspension, which can provide a sound basis for policy makers and fire-fighting authorities to plan and implement countermeasures. In the event of a large wildfire or a human-induced land surface disturbance, regardless of the reason for its occurrence, the speedy provision of accurate information to the general public is even more important.

To assess the hazards of air pollution after a large wildfire and to relate fire severity to changes in land cover, it is important to be able to detect the burned area accurately and rapidly. The amount of radionuclides released into the atmosphere by wildfires in the area around Chornobyl can vary greatly depending on the extent to which the fires spread^3,4,6,7^, because the radionuclides derived from the Chornobyl accident in the environment are known to be extremely heterogeneous^8–10^. For instance, Igarashi et al*.*^11^ found that the inventories of ^137^Cs and ^90^Sr at two sites located only 5 km apart differ significantly, by as much as 13 times and two times, respectively. The simplest way to estimate the burned area after a wildfire is to observe the site by conducting a human on-site survey. However, when a burned area extends over several hundred km^2^, it is almost impossible to investigate the site sufficiently by means of direct ground surveys. It would also be extremely difficult to identify and confirm all the locations of post-fire sites in cases where logistics are limited, radiation doses are dangerously high, and so on. Currently, ground surveying techniques utilizing terrestrial laser scanning^12,13^ and multiple satellites^14–17^ are available to detect burned areas. Free web services also provide information on burned areas^15–18^. However, these methods still have uncertainties^19^. It should also be noted that there is a time lag between the fire and the release of the corresponding burned area product. Even NASA’s MCD64, one of the most reliable burned area mapping algorithms currently available, takes two to three months to generate burned area products. The European Space Agency (ESA)’s burned area mapping algorithm FireCCI51 has not been updated since December 2020.

We consider that the main fundamental and methodological challenge underlying the uncertainty and delayed release of existing burned area products is the use of prescribed thresholds, which need be determined prior to detecting burned areas. Optimal thresholds are known to vary spatially due to differences in land cover and seasonality. Therefore, immediate and accurate detection of burned areas is challenging when applied to new target regions. In addition, parameter optimization requires accurate ground data that are usually impossible to collect immediately after a wildfire.

Currently, mid-resolution (500 m spatial resolution) remote sensing datasets can provide coarse-resolution global burned area products with a minimum detectable size of 25 ha (one pixel). Among the currently available global burned area data products, the Moderate Resolution Imaging Spectroradiometer (MODIS) MCD64 has so far been considered as the most accurate^20^. However, Zhu et al*.*^21^ validated the fire detection rate of MCD64 data product as approximately 50% for grassland and forest and only 1% for cropland in boreal Eurasia. Padilla et al*.*^22^ also found that MCD45 detected only 48% of the global burned area by using stratified random sampling and reference data. Additionally, the results gained using most of the regional burned area products indicate that the global products underestimate the actual burned area^23–25^.

On March 23, 2022, Ukraine’s regulatory authority informed the IAEA that firefighters were trying to extinguish wildfires in the Chornobyl Exclusion Zone (ChEZ)^26^. In order to assess the impact of the potential risk of radiation exposure from radionuclides resuspended and redistributed by the wildfires, it was necessary to accurately and rapidly identify the burned areas under the Russian invasion of Ukraine. The objective of this initiative was to detect burned areas near the ChEZ during the spring of 2022 that extend across wide areas accurately and rapidly without the need to perform field surveys or employ tuning thresholds. As a method to solve the problem of “using thresholds” to determine surface condition changes, we developed a tuning-free burned area detection algorithm (TuFda; Method S.I. 2.1) and made it available it on the free Google Earth Engine (GEE) cloud-based platform^27^. Originally, TuFda was designed to detect burned areas, but its characteristics make it capable of recognizing many kinds of land surface changes in addition to those caused by wildfires. The algorithm’s utilization on a free platform and its easy user-interface allows us access to information freely and easily.

## Results and discussion

### Impact of 2022 Chornobyl wildfires compared with previous fires

**Figure 1:**
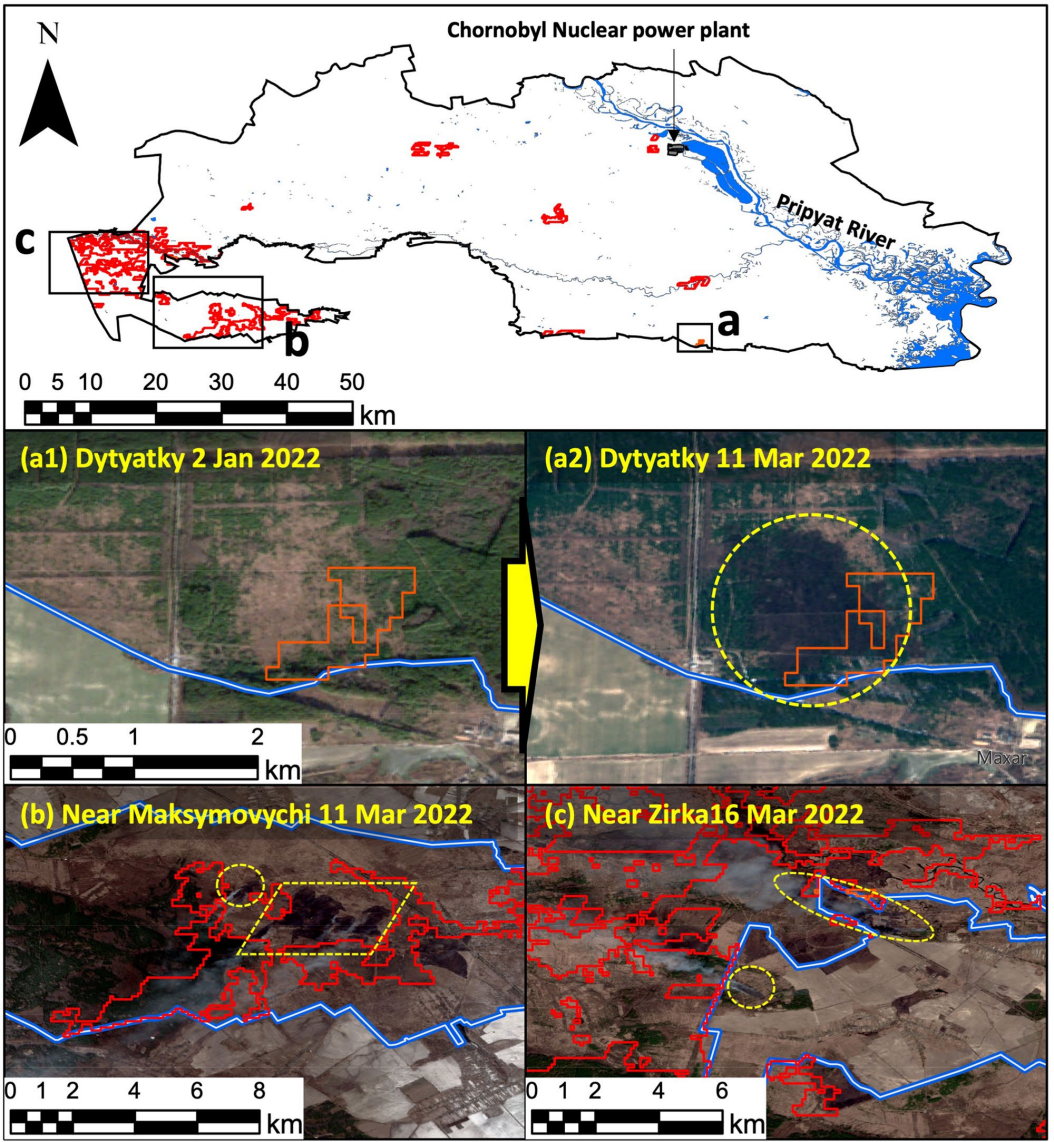
Detected burned area in February and March 2022 at the Chornobyl exclusion zone. Black solid indicates the border of Chornobyl exclusion zone. (**a1**) and (**a2**) show changes in the ground surface before and after the fire by Sentinel-2 (yellow dash line) at the place determined to be burned area in February 2022 (orange solid line). (**b**) and (**c**) show the actual wildfire areas and smokes confirmed by Sentinel-2 (yellow dash line) in west part of Chornobyl and where the burned areas were detected in March 2022 (red solid lines). The blue lines in (**a1**), (**a2**), (**b**) and (**c**) indicate the border of Chornobyl exclusion zone.

We anticipated that wildfires would occur in the Chornobyl area during the spring of 2022, and so we began detecting thermal anomalies with TuFda in February. The first thermal anomalies inside the ChEZ were detected during the period from 18 to 25 February. Then, the algorithm began to detect burned areas by identifying land surface differences between the pre-disturbance (3–17 February) and post-disturbance (26 February–11 March) periods. The total detected burned area in February was 0.4 km^2^ (Fig. 1). Regrinding the wildfire in February, the burned area near Dytyatky, a village in the southern part of the ChEZ, was confirmed using Sentinel-2 imagery. Figure 1a1 shows the true color surface on 2 January as determined from Sentinel-2 imagery (before fire), and Fig. 1a2 shows the true color surface on 11 March at the same location near Dytyatky after fire. TuFda detected a burned area to the south of the actual lost area. The second thermal anomalies were detected inside the ChEZ during the period from 11 to 21 March. The total detected burned area in March was 70 km^2^ (Fig. 1). The total burned area was significantly larger in March than in February. The largest of the burned areas in March were along the border of the western part of the ChEZ (Fig. 1b and 1c), and several major burned areas were also detected inside the ChEZ (Fig. 1). Protsak et al.^28,29^ used Sentinel-2 and visually identified the wildfires in the western part and inside the ChEZ during March 2022 based on their long experience of wildfires, and found the total burned area to be approximately 74 km^2^. This is the most reliable burned area estimate currently available. Interestingly, the locations and the total burned area detected by TuFda were almost identical to the results obtained by direct visual confirmation. Accordingly, we consider that TuFda can accurately replicate (or reproduce) the identification of wildfires in the ChEZ by skilled human observers. As of 13 June 2022, the date this paper was initially submitted, global burned area detection products obtained using algorithms such as MCD64A1 and/or FireCCI51 have not been published, and TuFda is currently the fastest automatic algorithm in the world at providing burned area estimates in terms of land surface disturbance in the ChEZ. In March 2022, wildfires were also detected in highly contaminated areas close to the Chornobyl nuclear power plant (Fig. 1). As Protsak et al*.*^28,29^ mentioned, the amount of radionuclides from the surface biomass resuspended into the atmosphere was considered to be limited, because the total burned area in March 2022 was smaller than that of the 2020 wildfires, resulting in little or no additional radiation exposure to the public^3–5^.

### Impact of the Russian invasion in northern Ukraine

**Figure 2:**
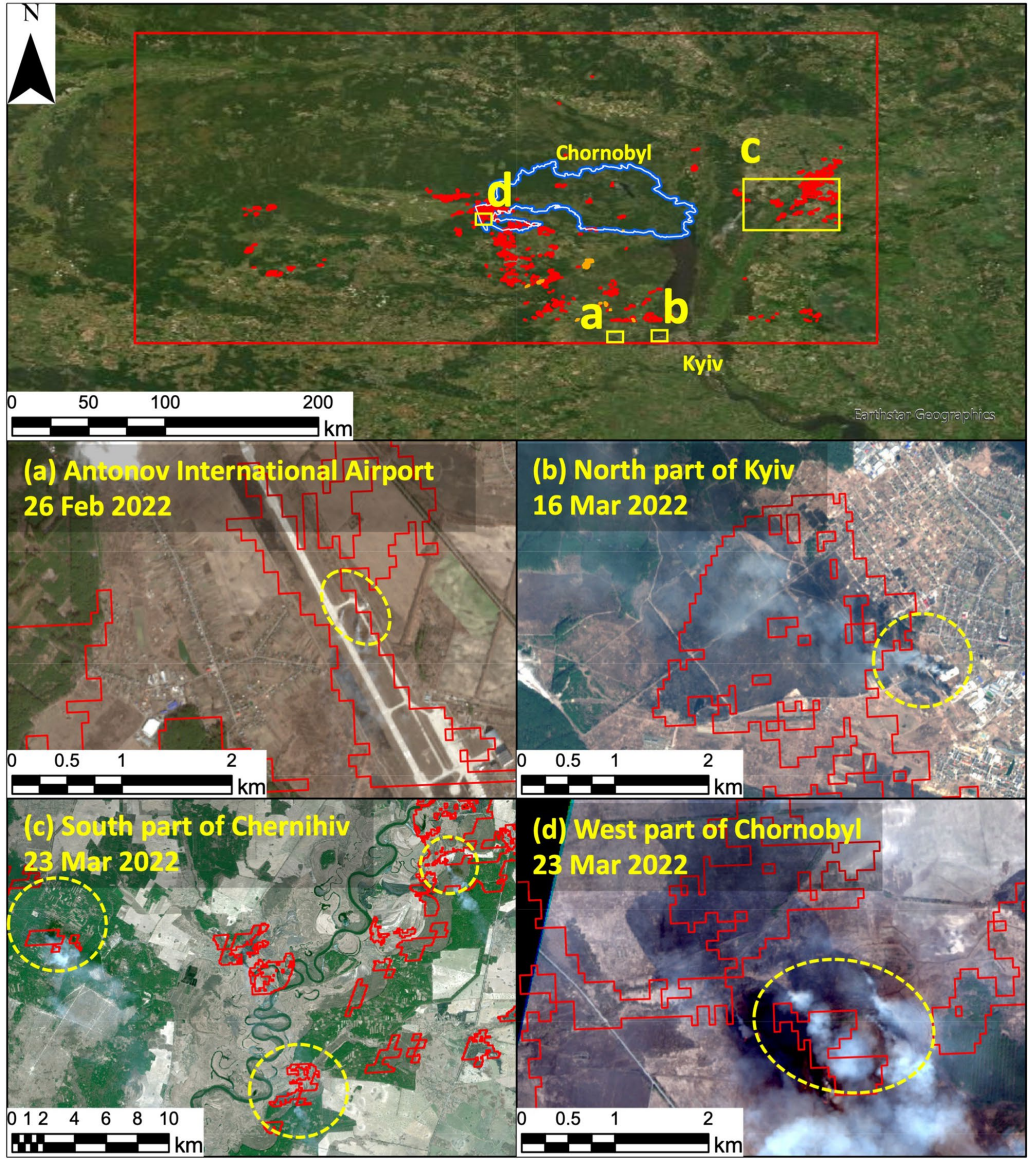
Detected burned area in February and March 2022 at the northern part of Ukraine. Orange and red line area indicate the detected burned area in February and March 2022, respectively. The blue lines indicate the border of Chornobyl exclusion zone. Red solid lines and yellow dash lines indicate the detected burned areas and the actual wildfire areas and smokes confirmed by Sentinel-2.

TuFda was developed to detect burned areas accurately and rapidly. We validated and confirmed its accuracy in identifying the burned areas that occurred in the ChEZ in 2015 and 2022 (SI 2.4). As was mentioned above, TuFda was able to replicate the results of human detection of burned areas caused by fire-related land cover changes that occurred in March 2022. TuFda, in principle, detects land surface changes over a period of interest. In February and March 2022, northern Ukraine was invaded by Russian forces. Fighting between Russian and Ukrainian forces would have affected forests, grasslands, and farmland in addition to military installations. We considered that TuFda could detect not only burned areas, but also other changes in land surface with a thermal anomaly. Accordingly, we extended the range of our algorithm to cover a wider area extending beyond the ChEZ (Fig. 2), in an effort to ascertain what was happening across northern Ukraine during February and March of 2022. Overall, TuFda detected some land surface changes within a fairly limited area in February (Fig. 2), while in March it detected land surface changes over a wider area.

We compared the burned area detected by TuFda with Sentinel-2 imagery for the period over which the fires were determined to have burned and provided some examples. For instance, a Sentinel-2 image taken on 26 February shows smoke at Antonov Airport, and this area was determined by TuFda to have been a burned area (Fig. 2a). A Sentinel-2 image taken on 16 March showed smoke and fire spreading in the northern part of Kyiv, and the same area was also identified by TuFda as a burned area (Fig. 2b). In Sentinel-2 imagery taken on 23 March, smoke and burned areas were found on both banks of the Desna River in southern Chernihiv (Fig. 2c) and in the western part of the ChEZ (Fig. 2d), and TuFda also identified the same areas as being burned areas. Locations where smoke and fire are spreading indicated by cloud-free Sentinel-2 imagery acquired during surmised period of wildfires were generally consistent with the areas identified by TuFda as burned areas. TuFda eventually detected a total burned area of 554 km^2^ in northern Ukraine during February and March 2022. We consider that the majority of the burned area detected by TuFda was burned in the course of the Russian invasion, which began on 24 February 2022.

In conclusion, we have developed a tuning-free burned area detection algorithm, and made it available on the free GEE platform, which allows it to be accessed from anywhere in the world. Our algorithm can identify burned areas with a two-week reference period before and after each incidence, and can provide burned area information two or three months earlier than previous “threshold-type” algorithms. Free and global accessibility, a user-friendly interface, and the rapid provision of accurate information on changes in land conditions combine to make this algorithm a highly useful tool that aids understanding of the environmental impacts of wildfires and/or wars, which in turn supports decision making and the implementation of post-event countermeasures and other activities. Again, the amount of radionuclides from the surface biomass resuspended into the atmosphere was considered to be limited, because the total burned area in March 2022 was smaller than that of the 2020 wildfires, resulting in little or no additional radiation exposure to the public.

## Method

The impacts of fire on vegetation detected by means of burned area detection tools vary depending on the type of fire, the fire’s behavior, and the time that elapses between the extinction of the fire and the acquisition of the image. In this study, we developed a tuning-free burned area detection algorithm that combines thermal anomaly detection using adaptive threshold (AT) and random forest (RF) optimization algorithms to identify burned areas. Initially, we employed the AT method to select pixels indicating potential burnout (details are available in SI 2). Our new tuning-free burned area detection algorithm was validated comparing its results against field observations (details are available in SI 1). We demonstrated that this tuning-free burned area detection algorithm is as accurate as existing algorithms and that its output can be published faster than that of existing algorithms (details are available in SI 2.4).

One may wonder whether the effect of snow cover changes the results of burned area in 2022. According to the truth color Sentinel-2 imagery in February 2022, we confirmed that the snow almost melted. In addition, the snow cover in the pre-wildfire in January 2022 were uniformly distributed around the fire mask. The calculated vegetation indices changed consistently after the pre-wildfire period. Consequently, the burned evidence would be insensitive to the snow cover, and mainly dependent on the changes in the vegetation indices after the wildfire.

## Supplementary Information

Supplementary Information.

## Data Availability

The burned area data were processed on the Google Earth Engine and a python platform. The script needed for calculating the vegetation indices and exporting the data can be found at https://code.earthengine.google.com/?scriptPath=users%2Fhujunjune%2FGEE%3ACEZ%2FTA%20RF%2Fexport%20rawdata%20%20to%20python. The tuning-free burned area algorithm were done using Python and can be accessed at link of python code.
