# Supplementary material for: Application of a tuning-free burned area detection algorithm to the Chornobyl wildfires in 2022
Source: Sci Rep. 2023 Mar 31;13:5236. doi: 10.1038/s41598-023-32300-5 (PMC10066350; doi:10.1038/s41598-023-32300-5)
Supplement: Supplementary file 1 — Supplementary Information. [file 41598_2023_32300_MOESM1_ESM.docx]

**Supplemental Information:** **Application of a tuning-free** **burned area detection algorithm** **to the Chornobyl wildfires in 2022**

**1. Study area**

The Chornobyl Exclusion Zone (ChEZ) is an area of approximately 2,600 km^2^ surrounding the Chornobyl Nuclear Power Plant (ChNPP), which was abandoned after the 1986 Chornobyl disaster. The ChEZ shares a 154.5 km international border with the Polesie State Radioecological Reserve to the north in Belarus. About 70% of the land is covered by boreal forest, which is dominated by Scots pine, and the remaining 30% is largely abandoned agricultural land^1,2^. The ChEZ is heavily contaminated by radionuclides and commercial use of the land has been prohibited. There has not been sufficient implementation of forest and fire management activities, which has led to overgrown vegetation, creating an environment susceptible to the outbreak and spread of wildfires.

Detailed field surveys have been conducted by local agencies in the ChEZ to quantitatively assess the impact of wildfires. In case of the large fires that occurred in 2015 and 2020, the burned areas were determined by actual field surveys. For the 2015 fire, we used the burned area survey data provided by the State Agency of Ukraine on Exclusion Zone Management Chornobyl Ecocentre. The extent of the burned area based on field surveys was 131 km^2^.

For the 2020 fire, in-situ surveys and visual observation of the burned areas in and around the ChEZ were conducted by the Ukrainian State Specialized Enterprise Pivnichna Pushcha (SI Fig. 1).

**
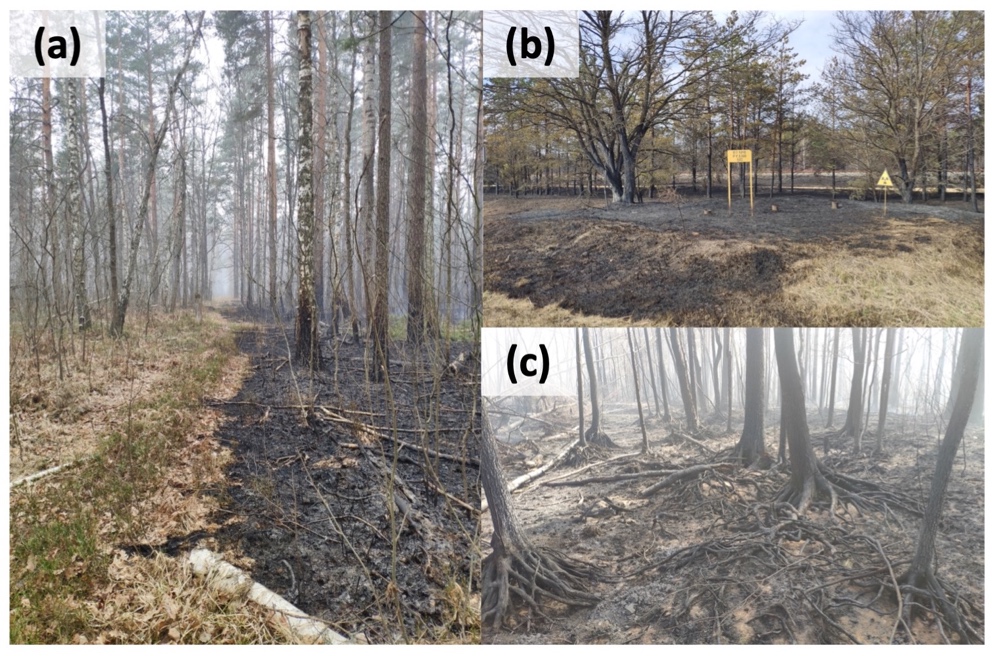
**

**Supplemental Information Figure 1:** Field surveys of the 2020 wildfires were conducted in the immediate aftermath of the fires. (a) The boundary between the burned and unburned areas near the village of Krasne (51.406° N, 29.739° E), 14 April 2020. (b) A wildfire also occurred in the Red Forest, which is the location of a temporary radioactive waste storage site (51.387° N, 30.077° E), 16 April 2020. (c) A wildfire mainly burned surface grass, litter and the organic soil layer, and then spread to the trunks and branches of trees in a forest in the northern part of the ChEZ (51.375° N, 29.704° E), 29 April 2020.

Regarding the 2020 wildfires, the Ukrainian State Specialized Enterprise Pivnichna Pushcha conducted actual field observations in the ChEZ. The zone is divided into seven forests. Each of these forests is subdivided into a large number of sections called “quartal (in Ukrainian)” (One quartal is typically 50**–**200 ha). Pivnichna Pushcha organized a radioecological dataset, including data on the total land area, total forest area, areas of coniferous forest, deciduous forest, and grassland (all species), water objects, other objects (roads, industrial buildings, etc.), and also burned areas after forest fires in a total of 1,810 quartals. A visual survey of the burned areas was conducted immediately after the April 2020 fires, in which researchers observed burned areas totaling over 553 km^2^ (SI Fig. 2).

**
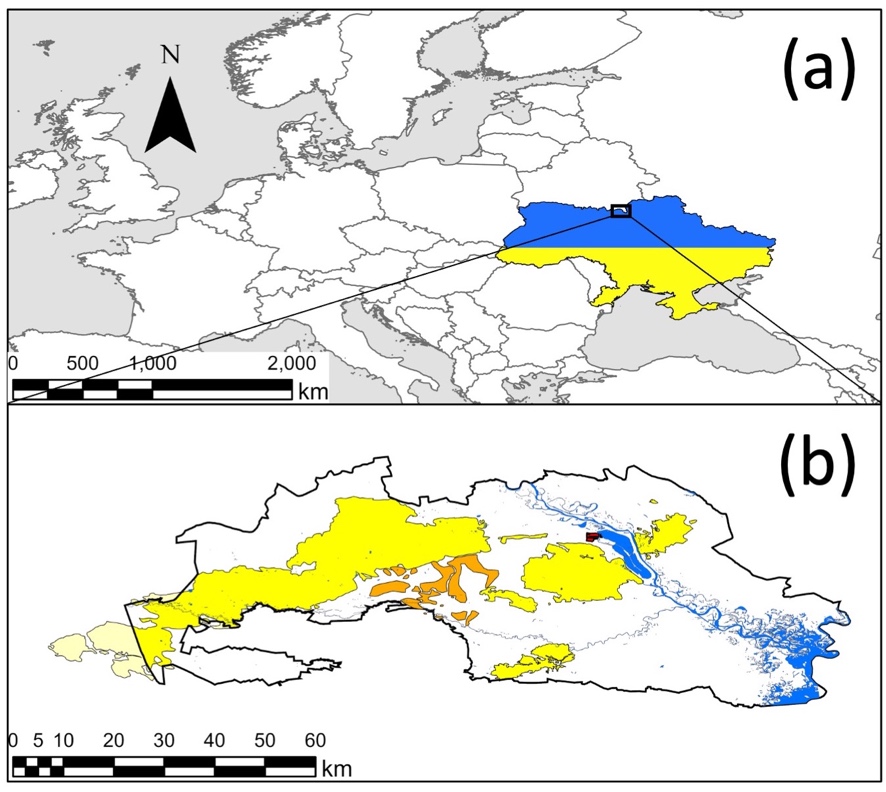
**

**Supplemental Information Figure 2:** Research area. (a) Location of the study area in Ukraine. (b) Burned areas in 2015 (orange), and in 2020 (yellow) according to field observations. Burned areas located outside of the ChEZ are shown in light-yellow color. Algorithm validation was performed for both the yellow and light-yellow areas

**2. Tuning-free wildfire detection algorithm**

The impacts of fire on vegetation are wide-ranging and contingent on the type of fire, the fire’s behavior, and the amount of time that has elapsed between the fire’s extinction and the image acquisition. Moreover, the post-fire signal as well as the manner in which it changes from the pre-fire reflectance, its temperature and its backscatter can vary widely. Accordingly, the evaluation of both the post-fire signal and the temporal changes in spectral behavior provide useful information for determining the fire’s impacts, while tracking post-fire changes over time contributes to determining vegetation regeneration patterns.

**2.1 Algorithm description**

In this study, we developed a tuning-free burned area detection algorithm that combines adaptive threshold (AT) detection and random forests (RF) optimization algorithms to identify burned areas. In the first step, we employed the adaptive threshold method to select potential burned pixels. To determine the adaptive thresholds for each vegetation index, the approximate radius $r$, which is the radius of a circle with the same area as the domain, of each potential burn scar was adopted to calculate specified regions in order to generate histograms.

$r={(A/\pi)}^{1/2}$ (1)

where $A$ is the area of each potential burn scar. A buffer with a radius *r* and its intersection with other buffers with radii of 1.1 *r* and 1.5 *r* were selected to generate the burned and unburned histograms, respectively. After normalization, we subtract the histogram of unburned area from that of burned area.

The cross point of subtracted histogram is the tuning-free ΔVIs_threshold_, namely, the threshold of the vegetation index. The potentially burned pixels were then estimated based on the following algorithm:

(NIR_post_ < NIR_threshold_) and (ΔVIs < ΔVIs_threshold_) (2)

where NIR_post_ is the surface reflectance of post-fire imagery and the ΔVIs represents the reflectance difference between the vegetation indices in the post- and pre-fire imagery. In the second step, we used the RF method to identify the coincident areas and optimize the accuracy of burned area detection. The potential burned pixels detected during the first step were adopted as the training masks to train the RF algorithm. After that, the trained RF algorithm was used to make the initial classification.

To improve the accuracy of burned area detection, we applied AT detections for seven categories of land cover with individual thresholds. This was because the more categories of land cover are used in the detection process, the more difficult it becomes to obtain sufficient contrast between burned and unburned areas to identify changes. Consequently, in the detection process, the land cover was combined into seven categories (water, grassland, cropland, coniferous forest, deciduous forest, shrubland, wetland, and others). AT detection was then carried out for each respective category of vegetation.

**2.2 Datasets and vegetation indices**

In this study, four commonly used vegetation indices derived from the MODIS were investigated as potential indicators: the Burned Area Index (BAI), Enhanced Vegetation Index (EVI), Normalized Burned Ratio (NBR), and Normalized Difference Vegetation Index (NDVI) (SI Table 1). Google Earth Engine (GEE) is a cloud-based remote sensing data processing and analysis platform that combines public access remote sensing and geospatial data with a highly efficient parallel computation service^3^. In the present study, we used GEE for the preprocessing, in particular with regard to image acquisition, water masks, and the derivation of vegetation indices.

**Supplemental Information Table 1:** Spectral indices evaluated in this study (NIR: Near Infrared, sSWIR: shorter short-wave infrared, lSWIR: longer short-wave infrared).

| **Index Full Name** | **Abbreviation** | **Equation** | **Reference** |
| --- | --- | --- | --- |
| Burned Area Index ^4^ | BAI | $\frac{1}{{(0.1-Red)}^{2}+{(0.06-NIR)}^{2}}$ | Chuvieco et al. 2002 |
| Enhanced Vegetation Index ^5^ | EVI | $\frac{2.5\times(NIR-Red)}{NIR+6.0\times Red-7.5\times Blue+1.0}$ | Huete et al. 2002 |
| Normalized Burn Ratio  ^6–8^ | NBR | $\frac{NIR-lSWIR}{NIR+lSWIR}$ | Koutsias et al. 2000  Key et al. 2006  García et al. 1991 |
| Normalized Difference Vegetation Index ^9^ | NDVI | $\frac{NIR-Red}{NIR+Red}$ | Tucker 1979 |

In burned area detection, the temporal interval between fire extinction and image acquisition is critical for detecting burned areas, especially in view of the rapid changes in vegetation and cloud cover. Since this burned area detection algorithm largely relies on the changes in vegetation indices, the acquisition of a consistent period of satellite images can extremely decrease the discrepancies of vegetation indices from satellite images getting from different periods moreover increase the performance of this algorithm. The combined constellation revisit frequencies of Sentinel-2 and Landsat-8 are 5 days and 16 days, respectively. If we consider the impact of the clouds on the acquisition of time-consistent images, these two datasets are difficult to fulfill our requirements.　Accordingly, in consideration of the timeliness and acquisition of the images, in this study, we mainly adopted the MODIS MOD09A1.006 and MYD09A1.006 (SI Table 2) to develop a rapid burned area detection algorithm (SI Fig. 3). These two surface reflectance datasets contain cloud, cloud shadow, and water masks, which can be used to mask out areas affected by clouds or water bodies.

**Supplemental Information Table 2:** Satellite data used in this study.

| **Data source** | **Acquisition Date** | **Image resolution (meter)** |
| --- | --- | --- |
| MOD09A1.006: Terra Surface Reflectance 8-Day Global 500m^41^ | Feb - June 2015  Feb - June 2020  Jan - May 2022 | 500 |
| MYD09A1.006: Aqua Surface Reflectance 8-Day Global 500m^42^ | Feb - June 2015  Feb - June 2020  Jan - May 2022 | 500 |
| MOD14A1.006: Terra Thermal Anomalies & Fire Daily Global 1km^43^ | April 2015  April 2020  Feb - Mar 2022 | 1000 |
| MYD14A1.006: Terra Thermal Anomalies & Fire Daily Global 1km^44^ | April 2015  April 2020  Feb - Mar 2022 | 1000 |
| Copernicus Global Land Cover Layers: CGLS-LC100 collection 3^45^ | 2015  2019 | 100 |


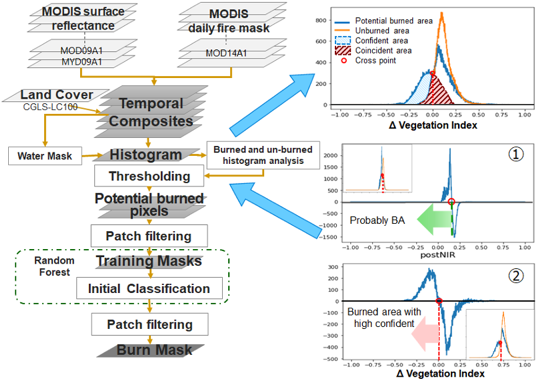


**Supplemental Information Figure 3: A flowchart of the tuning-free algorithm.**

We used the MODIS MOD14A1 to acquire the fire start and finish dates and information on potentially active fires during the detection period. Then we automatically composited and calculated the images with the pre-fire and post-fire vegetation indices. The non-overlapping adjacent sliding temporal windows (8 weeks) of the two MODIS 8-day surface reflectance products were adopted to composite the imagery. Post-fire imagery acquired within 15 days following the fire finish date was adopted to calculate the post-fire vegetation indices. Imagery acquired within the 15 days prior to the fire start date was adopted to calculate the pre-fire vegetation indices in order to grasp the changes in the vegetation indices and minimize any seasonal differences between the pre- and post-fire vegetation indices.

**2.3 Accuracy assessment**

The accuracy assessment is based on a confusion matrix to calculate the assessment indices. The parameters of the confusion matrix are obtained from the sampled pixels within the boundaries of the ChEZ and utilize available products (classified data) and observations (reference data). In the confusion matrix, the detected pixels are coded as "burned" and the rest of the pixels are coded as "unburned". The confusion matrix is a square matrix consisting of two rows and two columns that provides a comparison of the burned and unburned data (SI Table 3). The confusion matrix cell entries for pixels are based on the ratio of the areas of agreement and disagreement in the ChEZ. *P_ij_* represents the summation of all interpretable pixels divided by the number of pixels for the region of interest. The accuracy indicator in this study includes the overall accuracy (OA),

$OA =P_{11}+P_{12}$ (3)

Kappa accuracy (KA),

$KA=\frac{OA-P_{e}}{1-P_{e}}$ (4)

where $P_{e}=P_{0+}P_{+0}+P_{1+}P_{+1}$

commission error ratio (CE),

$CE={P_{10}}/{P_{1+}}$ (5)

omission error (OE),

$OE={P_{01}}/{P_{+1}}$ (6)

To evaluate the burned area detection accuracy of the products, the critical success index (CSI) commonly used in forecast verification is introduced into burned area accuracy assessment to measure the burned area detection performance and summarize it in a single metric.

$CSI =\frac{P_{11}}{P_{11}+P_{10}+P_{01}}$ (7)

**Extended Data Table 3 | The confusion matrix for the accuracy assessment. *P*_ij_ is expressed as the ratio of the area of agreement (diagonal cells) and disagreement (off-diagonal cells).**

|  | Reference Data | | |
| --- | --- | --- | --- |
| Classified Data | Unburned | Burned | Total |
| Unburned | *P*_00_ | *P*_01_ | *P*_0+_ |
| Burned | *P*_10_ | *P*_11_ | *P*_1+_ |
| Total | *P*_+0_ | *P*_+1_ | 1 |

**2.4 Evaluation of the tuning-free burned area detection algorithm**

Utilizing our burned area detection algorithm, we extracted and evaluated burned areas with respect to the observation data for 2015 and 2020. These observation data were obtained and mapped in the course of field surveys conducted in 2015 and 2020 after wildfire incidents. In a statistical test, MCD64A1 was identified as the most accurate of the existing global burned area products, followed by FireCCI51^10^. Then we compared our burned area product against those of MCD64A1 and FireCCI51 (SI Fig. 4 and 5). Of the moderate-resolution burned area products, FireCCI51 utilizes MODIS 250-m resolution surface reflectance data, and MCD64A1, like our algorithm, utilizes MODIS 500-m resolution surface reflectance data to detect burned areas. According to the accuracy assessment, FireCCI51’s product had the highest OA due to its finer spatial resolution, while our algorithm, which is an AT & RF algorithm, had the highest overall Kappa accuracy and critical success index. The Kappa accuracy is a measure of how the classification results compared to the values assigned by chance, which is a useful indicator for evaluating problems involving an imbalance between the classes. Our AT & RF algorithm significantly outperformed the existing algorithms, exhibiting improvements in Kappa accuracy of 7.4% and 6.9% compared to MCD64A1 for 2015 and 2020, respectively, and of 1.5% in total compared to FireCCI51. The accuracy of the critical success index showed that the burned area accuracy of our AT & RF algorithm is 6.9% and 2.1% higher on average than that of MCD64A1 and FireCCI51, respectively. Taken together, the accuracy of all three indicators showed that our burned area detection algorithm has a superior ability to identify burned areas. In terms of error assessment, this algorithm reduced the omission error ratio by an average of 16.9 % and 13.3 % compared to that of MCD64A1 and FireCCI51, respectively, while achieving the same commission error ratio as MCD64A1, which was only 0.8 % higher than that of FireCCI51. These results demonstrated the superiority of our AT & RF algorithm in reducing errors in identifying burned areas in the ChEZ. Compared with the field observation, a notable omission happened on the edge of the northwest of the burned area for all burned area products. This is because of the low intensity of the fire in these regions. When only the timber and the grass were burnt, all trees are alive and no significant changes can be detected by vegetation indices. Consequently, it is difficult to detect fire scars from the satellite images with the relative coarse spatial resolution.


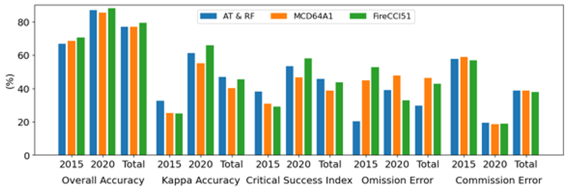


**Supplemental Information Fig. 4: Comparison of burned area detection accuracy in the ChEZ.** Our AT & RF algorithm uses the adaptive threshold method based on the random forest algorithm. MCD64A1 and Fire CCI51 are open dataset algorithms issued by NASA and the European Space Agency (ESA), respectively.


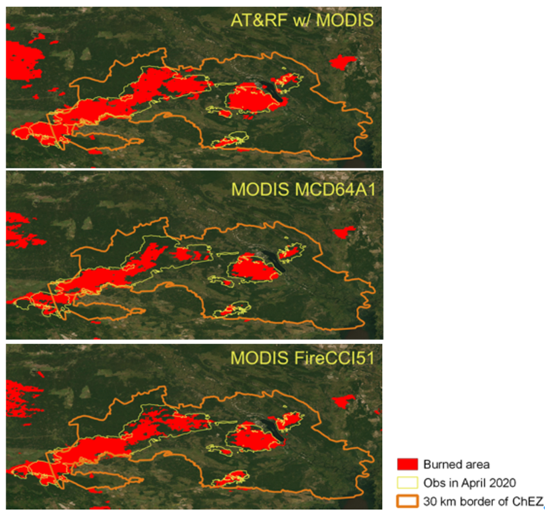

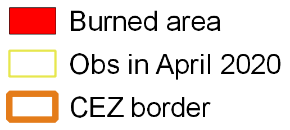


**Supplemental Information Fig. 5: The spatial distribution of wildfires between 8 and 24 April 2020.**

The red-colored regions represent estimated burned areas. The regions surrounded by yellow lines represent burned areas obtained through field surveys. The orange line shows the border of the ChEZ.

**References**

1. ﻿Yoschenko, V., Kashparov, V. & Ohkubo, T. Radioactive Contamination in Forest by the Accident of Fukushima Daiichi Nuclear Power Plant: Comparison with Chernobyl. in Radiocesium Dynamics in a Japanese Forest Ecosystem 3–22 (Springer Singapore, 2019). doi:10.1007/978-981-13-8606-0_1.
2. ﻿Holiaka, D. et al. Scots pine stands biomass assessment using 3D data from unmanned aerial vehicle imagery in the Chernobyl Exclusion Zone. J. Environ. Manage. 295, 113319 (2021).
3. ﻿Gorelick, N., Hancher, M., Dixon, M., Ilyushchenko, S., Thau, D., & Moore, R. (2017). Google Earth Engine: Planetary-scale geospatial analysis for everyone. Remote Sensing of Environment, 202, 18–27. <https://doi.org/10.1016/j.rse.2017.06.031>
4. Chuvieco, E., Martín, M. P. & Palacios, A. Assessment of different spectral indices in the red-near-infrared spectral domain for burned land discrimination. *International Journal of Remote Sensing* **23**, 5103–5110 (2002).
5. Huete, A. *et al.* Overview of the radiometric and biophysical performance of the MODIS vegetation indices. *Remote Sensing of Environment* **83**, 195–213 (2002).
6. Koutsias, N. & Karteris, M. Burned area mapping using logistic regression modeling of a single post-fire Landsat-5 Thematic Mapper image. *International Journal of Remote Sensing* **21**, 673–687 (2000).
7. Key, C. H., Benson, N. C. & USDA Forest Service, R. M. R. S. *Landscape Assessment: Ground measure of severity, the Composite Burn Index; and Remote sensing of severity, the Normalized Burn Ratio*. http://pubs.er.usgs.gov/publication/2002085 (2006).
8. García, M. J. L. & Caselles, V. Mapping burns and natural reforestation using thematic Mapper data. *Geocarto International* **6**, 31–37 (1991).
9. Tucker, C. J. Red and photographic infrared linear combinations for monitoring vegetation. *Remote Sensing of Environment* **8**, 127–150 (1979).
10. Padilla, M. et al. Comparing the accuracies of remote sensing global burned area products using stratified random sampling and estimation. Remote Sensing of Environment 160, 114–121 (2015).
